# Supplementary figures and images for: The liprin-α/RIM complex regulates the dynamic assembly of presynaptic active zones via liquid–liquid phase separation
Source: PLoS Biol. 2025 Jun 10;23(6):e3002817. doi: 10.1371/journal.pbio.3002817 (PMC12151379; doi:10.1371/journal.pbio.3002817)

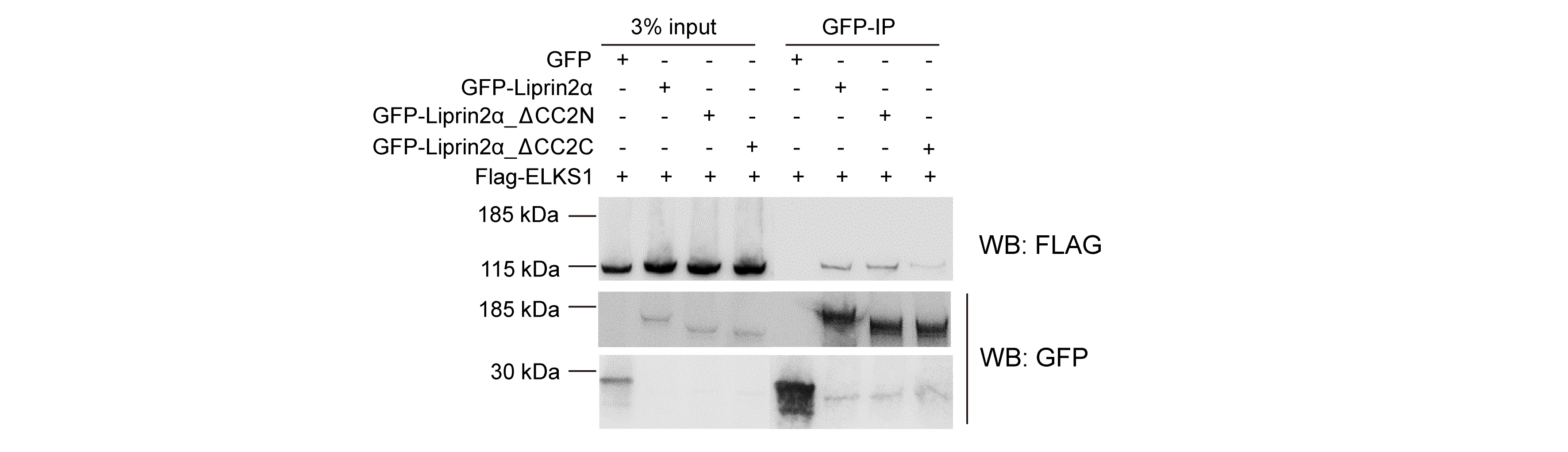

Supplement: S1 Fig — GFP-tagged liprin-α2 variants were co-expressed with Flag-tagged ELKS1 in HEK293T cells. The result indicates the CC2N fragment is essential for the binding of liprin-α2 to ELKS1. The data underlying this figure can be found in S1 Raw Images. (TIF) [file pbio.3002817.s001.tif]

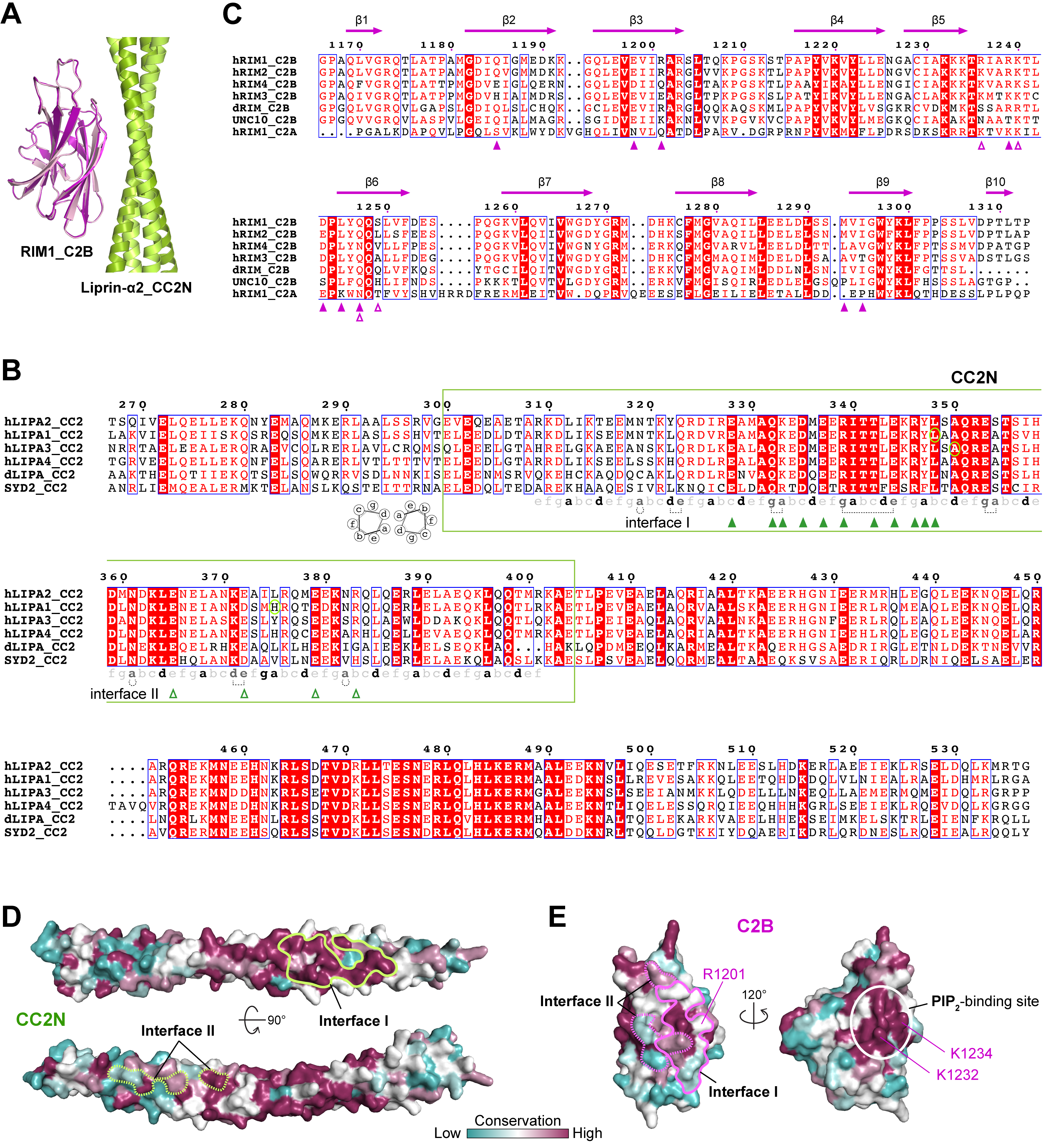

Supplement: S2 Fig — (A) Structural comparison of the two C2B molecules (e.g., C2B and C2B′ in Fig 2A) in the CC2N/C2B tetrameric complex. The corresponding interfaces I on CC2N are aligned well, indicating the symmetric binding of C2B to the dimeric CC2N coiled coil. (B) Multisequence alignment of the CC2 segment from liprin-α family proteins. Species abbreviations: ‘h’ for human, ‘d’ for Drosophila, and SYD2 as the Caenorhabditis elegans liprin-α homolog. Heptad repeats of coiled-coil structures in the CC2N sequence are annotated, with heptad labels in black and dark gray denoting residues involved in coiled-coil formation through hydrophobic and polar interactions, respectively. Polar interactions are indicated by dashed lines, while residues involved in interfaces I and II are marked by solid and open purple triangle-ups. Mutation sites associated with neurodevelopmental disorders are encircled. (C) Multisequence alignment of the C2B domains from RIM family proteins. The sequence of the C2A domain in human RIM1 was also included in the alignment for comparison. UNC10 is a RIM homolog in C. elegans. Residues involved in interfaces I and II are indicated by solid and open green triangle-ups, respectively. (D) Surface conservation analysis of the CC2N coiled-coil structure. Conservation scores for each residue were calculated based on the alignment in panel B. (E) Surface conservation analysis of the C2B structure. Conservation scores for each residue were calculated based on the alignment in panel C. The highly conserved PIP2-binding site is indicated by a circle. (TIF) [file pbio.3002817.s002.tif]

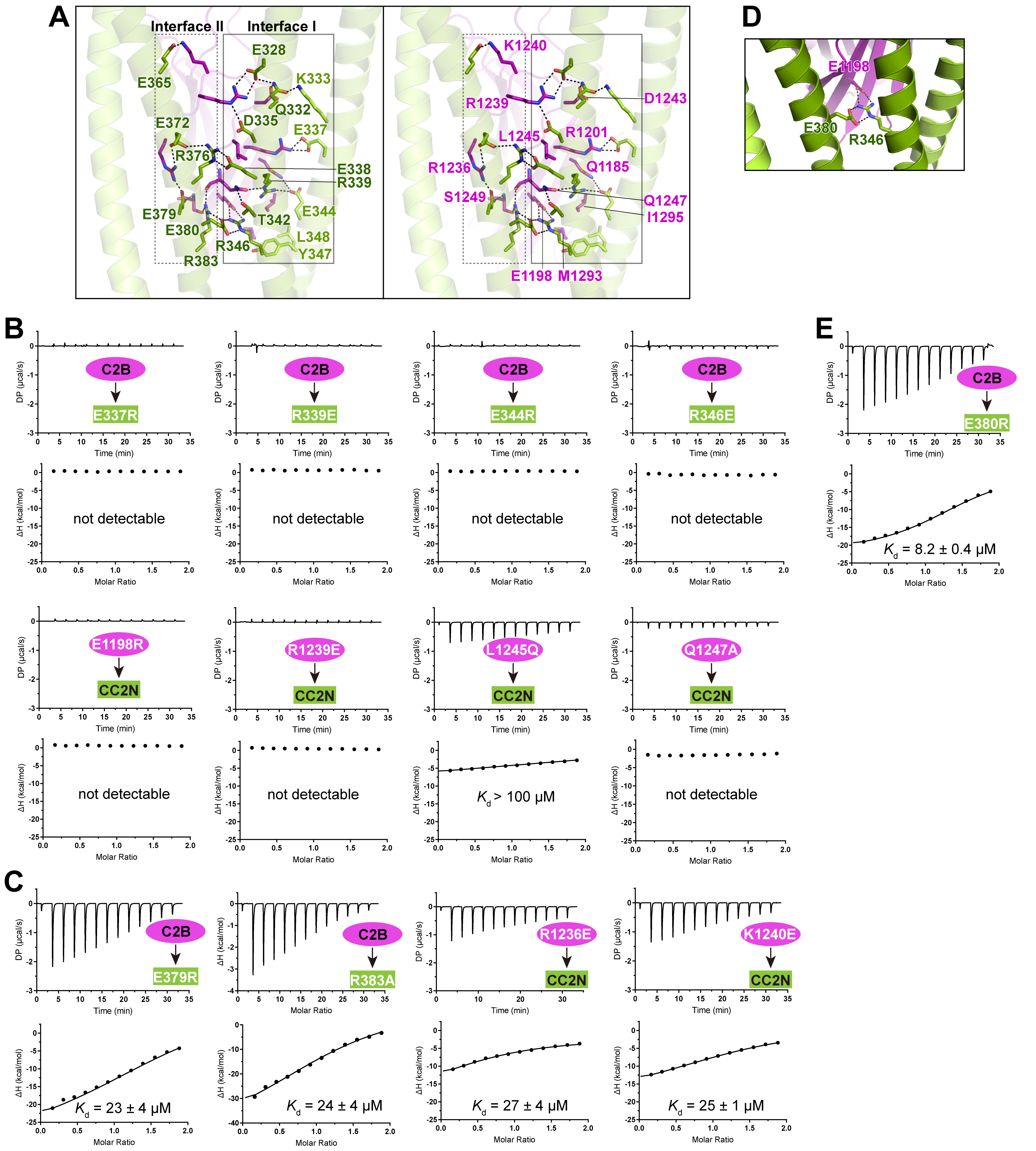

Supplement: S3 Fig — (A) Stereo view showing the molecular details of the coupling between the two interfaces of CC2N and C2B. Key salt bridges and hydrogen bonds are indicated by dashed lines. (B) ITC-based analyses of interface I mutation effects on the CC2N/C2B interaction. (C) ITC-based analyses of interface II mutation effects on the CC2N/C2B interaction. (D) Structural analysis of two interacting CC2N coiled coils at interface I. The salt bridge formed between R346liprin-α2 and E1198RIM1 is stabilized by E380liprin-α2. (E) ITC-based analysis showing the mild disruptive effect of the E380R mutation in CC2N on the CC2N/C2B interaction. The data underlying panels B, C and E can be found in S1 Data. (TIF) [file pbio.3002817.s003.tif]

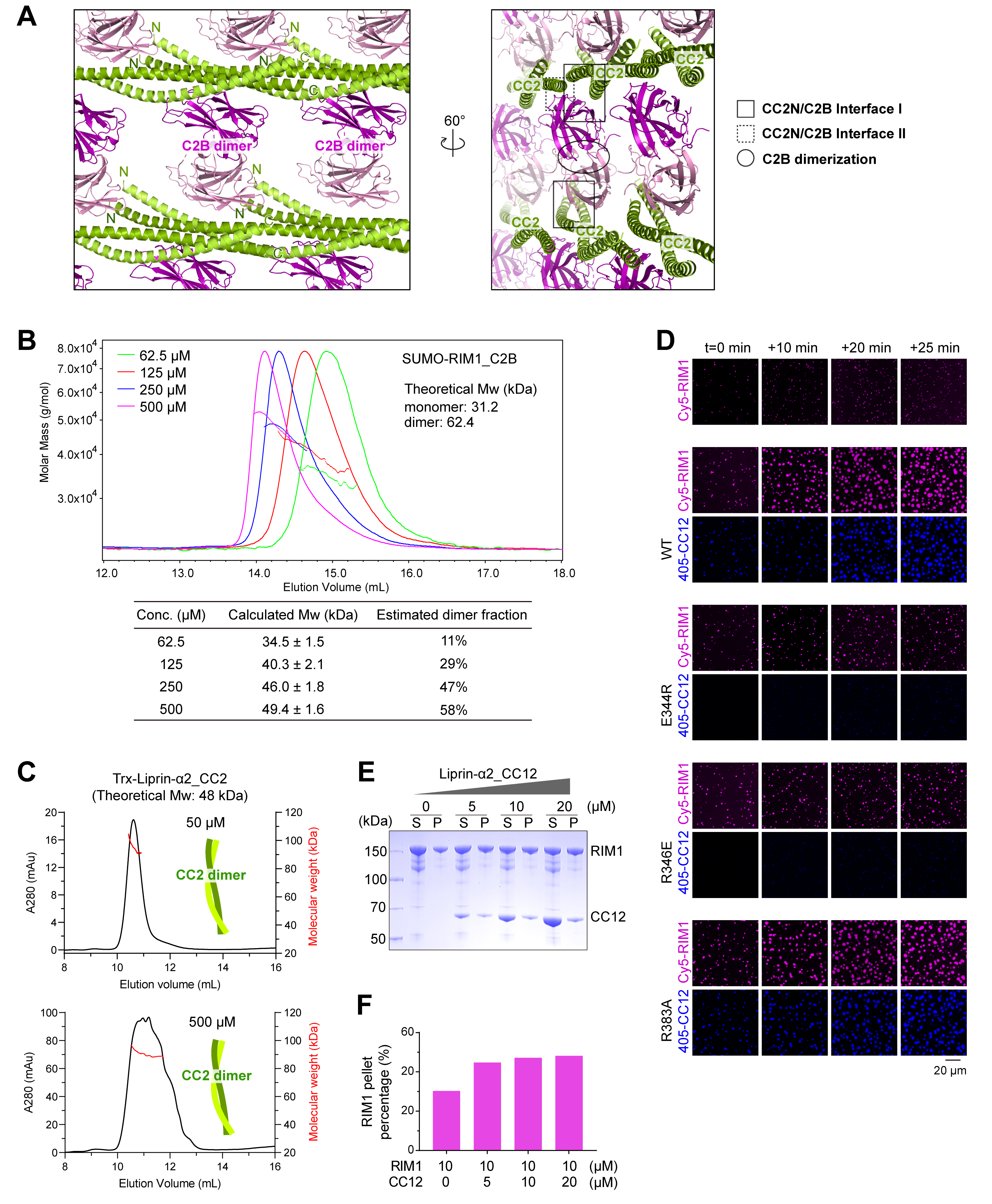

Supplement: S4 Fig — (A) Crystal packing analysis of the liprin-α2_CC2N/RIM1_C2B complex. Different intermolecular interfaces are highlighted. (B) Dimerization fraction analysis of RIM1_C2B in solution using aSEC coupled with MALS. (C) Molecular weight analysis of liprin-α2_CC2 in solution using aSEC coupled with MALS. (D) The time course of condensate formation following the mixing of RIM1 and liprin-α2_CC12 or its mutants. The concentration of each protein within the mixture was 5 µ M. (E) Sedimentation-based assay indicating the distribution of RIM1 full-length protein in the supernatant (S) and pellet (P) when mixed with increasing concentrations of liprin-α2_CC12. (F) Quantification of the RIM1 content of pellet fraction of samples shown in panel E. The data underlying panels B, C, E, and F can be found in S1 Data and S1 Raw Images. (TIF) [file pbio.3002817.s004.tif]

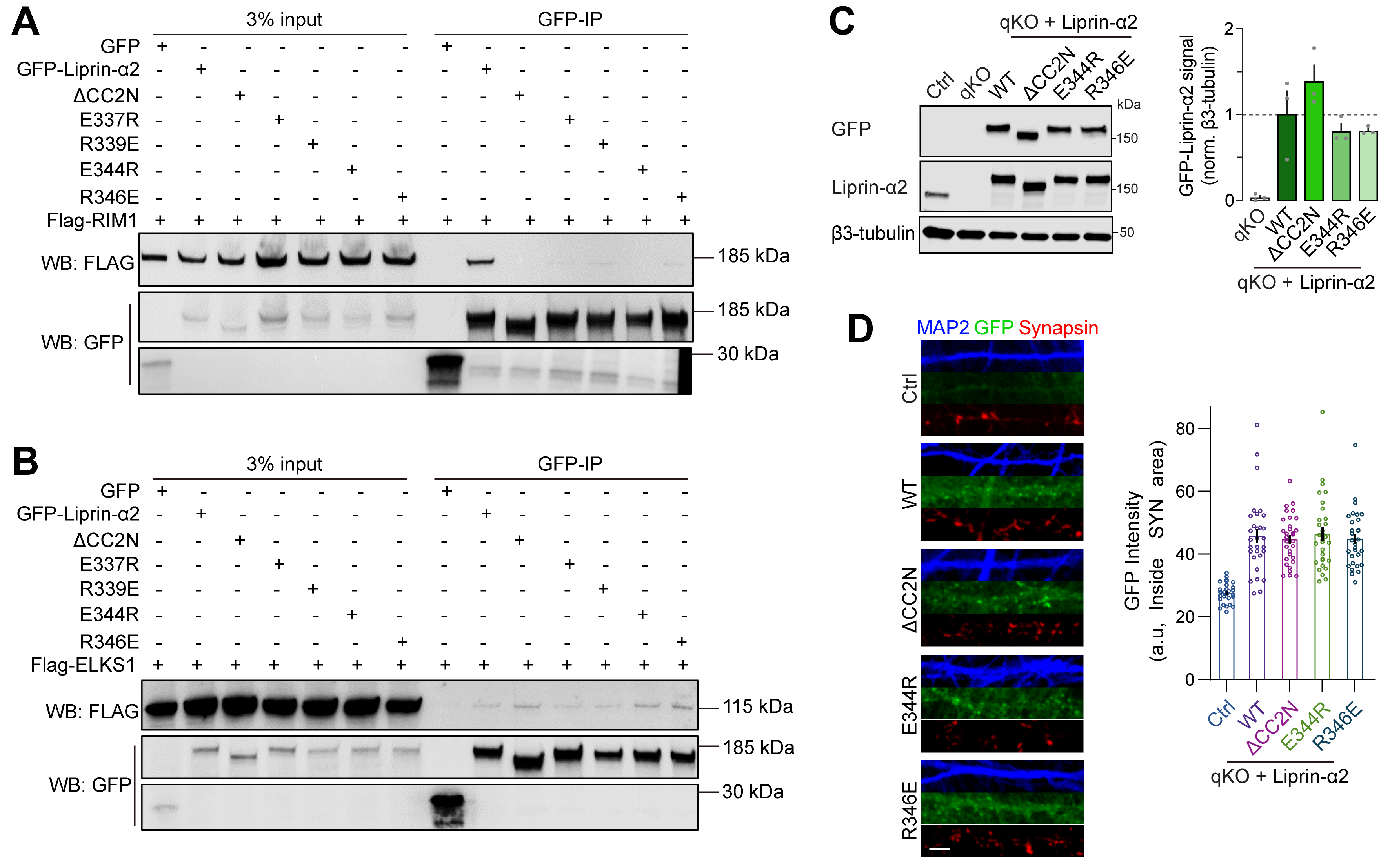

Supplement: S5 Fig — (A) Co-immunoprecipitation analysis of the binding of liprin-α2 variants to RIM1. Consistent with our ITC-based analyses (S3B Fig), the interface I mutations in liprin-α2 disrupt the liprin-α2/RIM1 interaction. Results are repeated by three independent batches of experiments. (B) Co-immunoprecipitation analysis of the binding of liprin-α2 variants to ELKS1. The interface I mutations, especially E344R and R346E in liprin-α2, showed minimal interference with ELKS1 binding. Thus, these mutations were selected for the following rescue assays in liprin-α qKO neurons. (C) western blot analysis confirming comparable expression levels of liprin-α2 variants expressed by lentivirus transduction in liprin-α qKO neurons. (D) Quantifications of endogenous GFP fluorescence levels within nerve terminals (co-localized with Synapsin signals) in control (wild-type, no GFP expression) conditions, as well as in Liprin-α qKO cells transduced with lentiviruses expressing either liprin-α2-GFP WT or its mutants (ΔCC2N, E344R, and R346E). The data underlying this figure can be found in S1 Data and S1 Raw Images. (TIF) [file pbio.3002817.s005.tif]

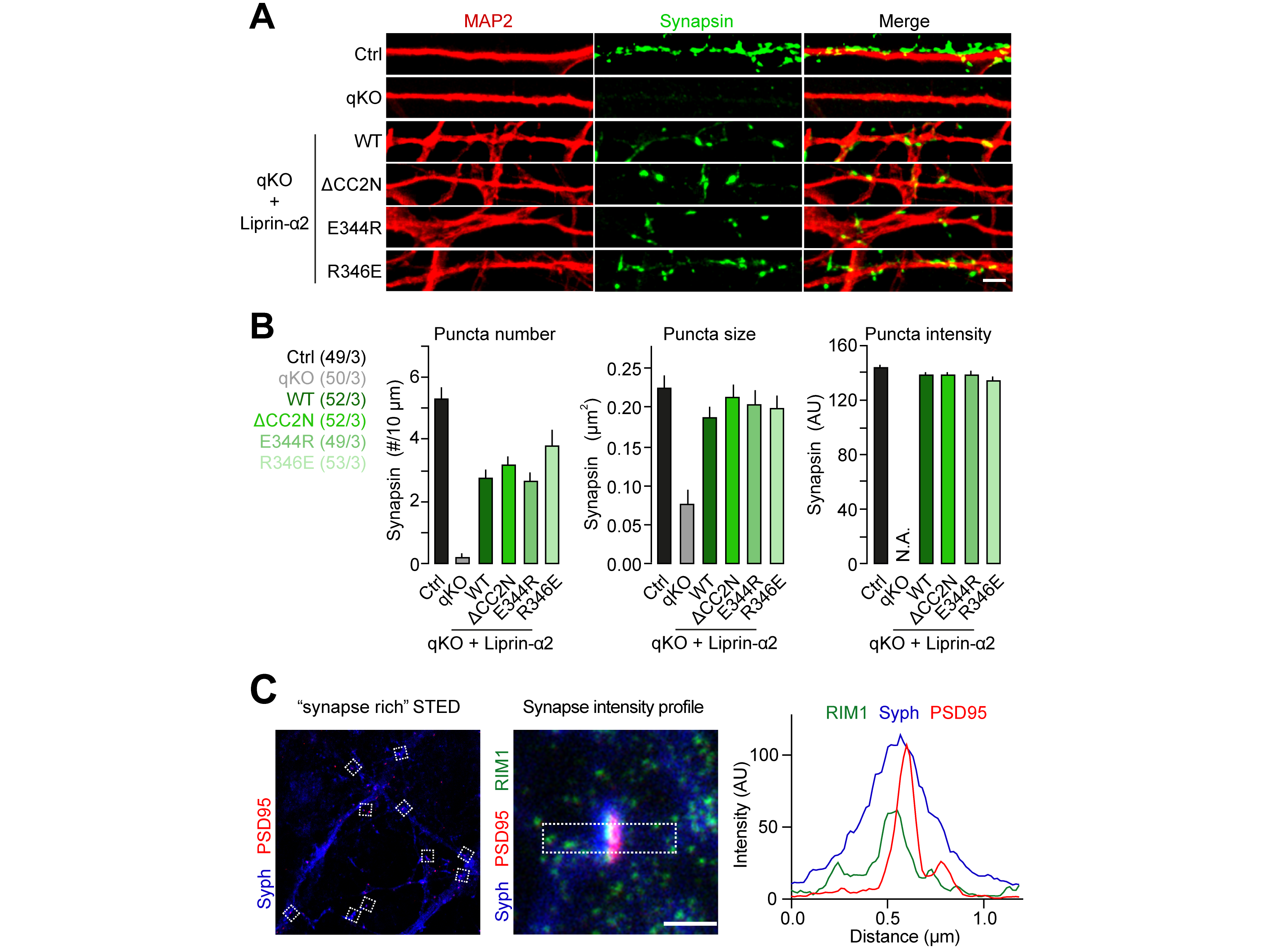

Supplement: S6 Fig — (A) Representative confocal micrographs showing presynaptic synapsin (green) puncta closely apposed to dendritic MAP (red) profiles under the indicated conditions. Scale bar: 5 μm. (B) Quantification of synapsin puncta density (left), size (middle), and intensity (right). The data reflects the minimal impact of liprin-α/RIM complex perturbations on synapse morphological integrity. The number of cells/batches analyzed for each condition is indicated on the right. (C) Microscopic analysis to identify synapses and measure the sub-synaptic distribution of presynaptic proteins using STED microscopy. Synapse-rich regions identified by Synapsin/PSD95 appositions were visualized at low magnification (left), followed by high magnification imaging (middle), with signal intensity quantified relative to the distance from postsynaptic PSD95 signals (right). The data underlying panels B and C can be found in S1 Data. (TIF) [file pbio.3002817.s006.tif]

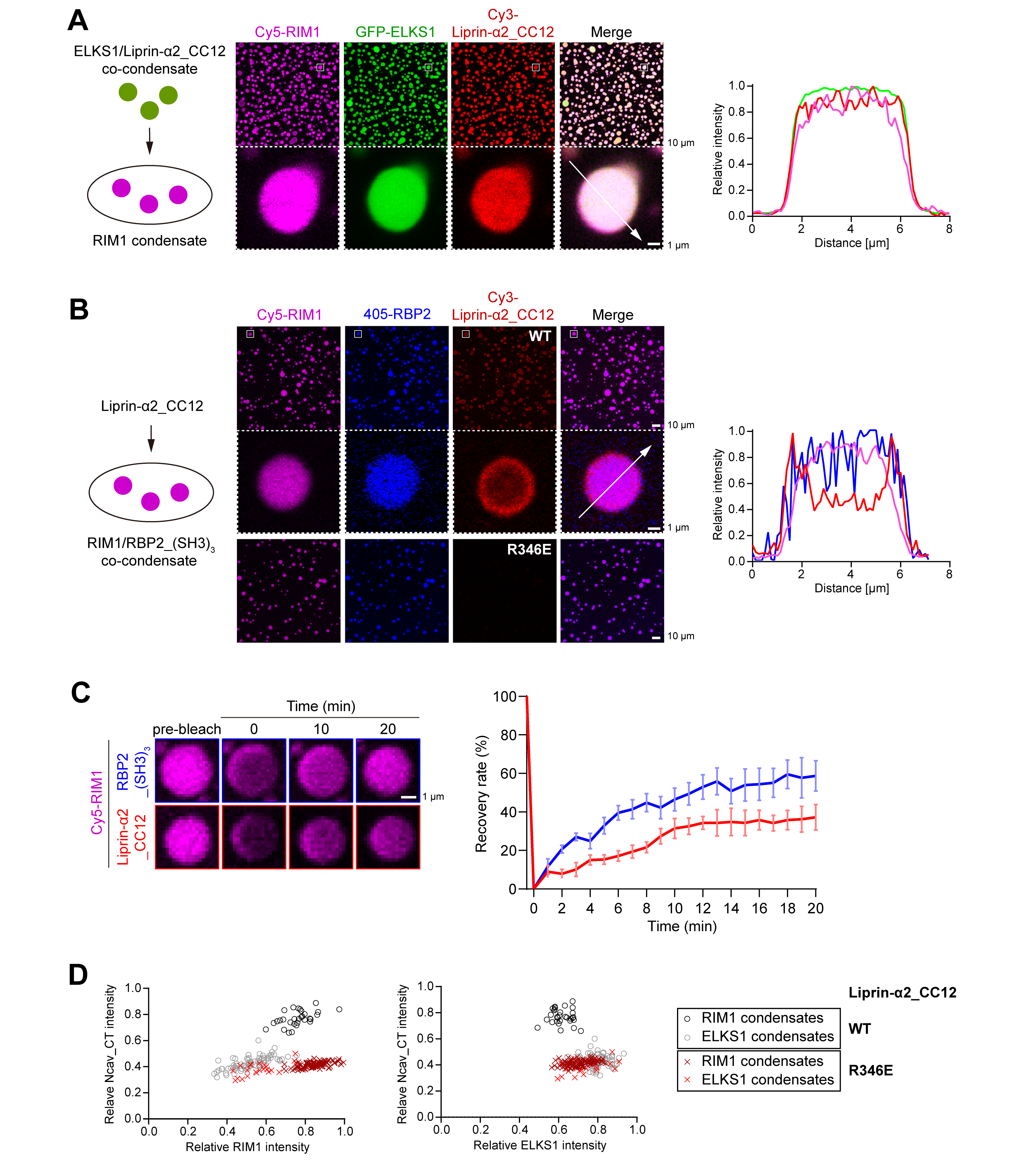

Supplement: S7 Fig — (A) Confocal imaging analysis of the LLPS mixture containing ELKS1/liprin-α2_CC12 and RIM1 condensates. A magnified view of a representative droplet is displayed below, with a line analysis of fluorescence signal intensities along the indicated line. The concentration of each protein was 5 μM. (B) Confocal imaging analysis of RIM1/RBP2 co-condensates upon adding liprin-α_CC12 WT or the R346E mutant. A magnified view of a representative droplet, as boxed in the WT condition, is displayed below, with a line analysis of fluorescence signal intensities along the line. The concentration of each protein was 5 μM. (C) FRAP analysis of RIM1 condensates in the presence of RBP2_(SH3)3 or liprin-α_CC12. (D) Plot analyses illustrating the intensity relationship between NCav_CT and RIM1 (left panel) or ELKS1 (right panel) fluorescence signals in RIM1 or ELKS1 condensates in the presence of liprin-α_CC12 WT or R346E. The data underlying this figure can be found in S1 Data. (TIF) [file pbio.3002817.s007.tif]

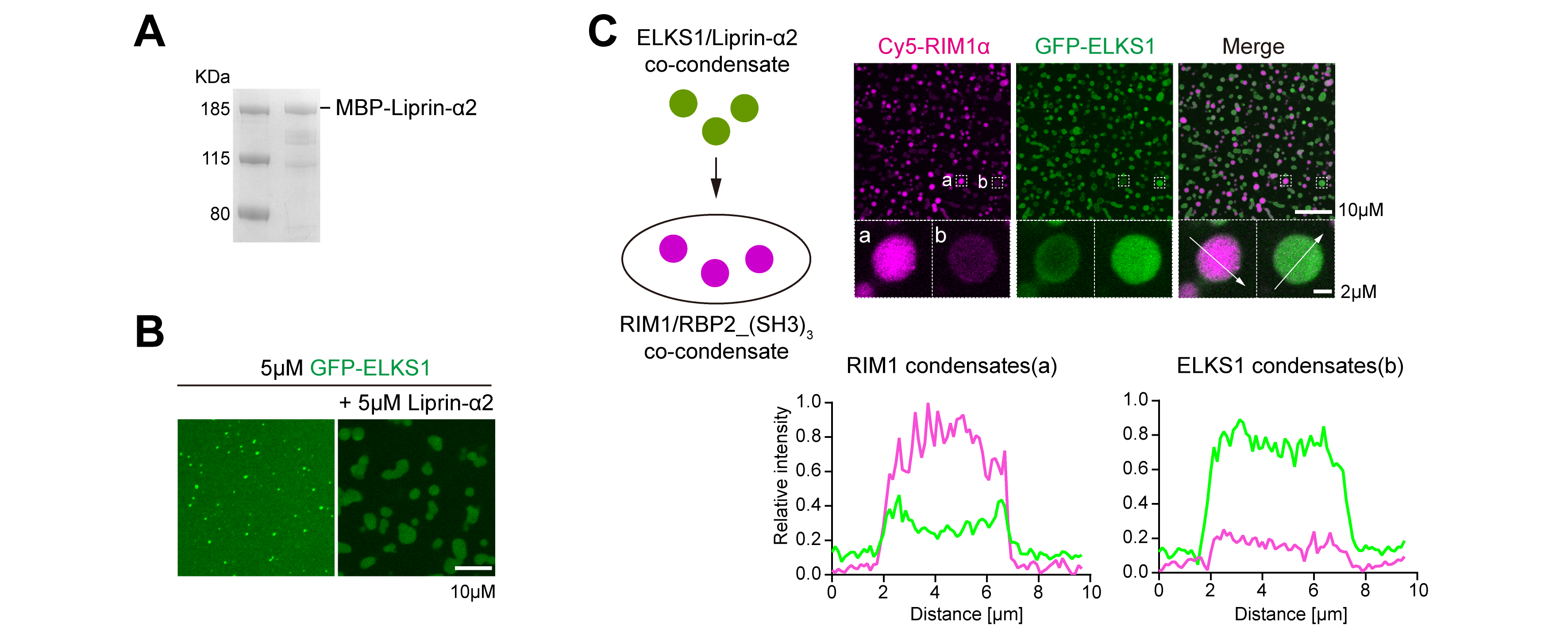

Supplement: S8 Fig — (A) SDS-PAGE analysis of purified full-length liprin-α2. (B) Confocal imaging of ELKS droplets with or without liprin-α2. (C) Confocal imaging analysis of the LLPS mixture containing the ELKS1/Liprin-α2 co-condensate and the RIM1/RBP2_(CH3)3 co-condensate. Magnified views of representative RIM1(a) and ELKS1(b) droplets were displayed below, with line analyses of fluorescence signal intensities along the indicated lines. The concentration of each protein within the mixture was 5 µ M. The data underlying panels A and C can be found in S1 Data and S1 Raw Images. (TIF) [file pbio.3002817.s008.tif]
