# Supplementary material for: The liprin-α/RIM complex regulates the dynamic assembly of presynaptic active zones via liquid–liquid phase separation
Source: PLoS Biol. 2025 Jun 10;23(6):e3002817. doi: 10.1371/journal.pbio.3002817 (PMC12151379; doi:10.1371/journal.pbio.3002817)
Supplement: S1 Text — Table A: X-ray data collection and refinement statistics. Table B: Summary of plasmids. Table C: Antibody identifiers. (DOCX) [file pbio.3002817.s009.docx]

**Table A. X-ray data collection and refinement statistics.**

| **Data collection** |  |  |
| --- | --- | --- |
| Space group | *P* 2_1_ |  |
| Cell dimensions |  |  |
| *a*, *b*, *c* (Å) | 58.663, 92.697, 66.583 |  |
| α, β, γ (°) | 90, 102.95, 90 |  |
| Resolution (Å) | 50–2.75 (2.8-2.75) |  |
| *R*_merge_^a^ | 0.149 (1.151) |  |
| *I*/σ*I* | 13.8 (1.4) |  |
| *CC*_1/2_^b^ | 0.995 (0.714) |  |
| Completeness (%) | 98.9 (98.0) |  |
| Redundancy | 6.3 (5.9) |  |
| **Refinement** |  |  |
| Resolution (Å) | 50-2.75 (2.92-2.75) |  |
| No. reflections | 17881 (2950) |  |
| *R*_work_ / *R*_free_^c^ | 0.205 (0.290) / 0.234 (0.323) |  |
| No. atoms |  |  |
| Protein | 3832 |  |
| Ligand/ion | 0 |  |
| Water | 20 |  |
| Mean *B* (Å) |  |  |
| Protein | 68.8 |  |
| Ligand/ion | - |  |
| Water | 58.9 |  |
| r.m.s. deviations |  |  |
| Bond lengths (Å) | 0.002 |  |
| Bond angles (°) | 0.5 |  |
| Ramachandran analysis |  |  |
| Favored region (%) | 98.7 |  |
| Allowed region (%) | 1.3 |  |
| Outliers (%) | 0 |  |
| The numbers in parentheses represent values for the highest resolution shell. | | |
| ﻿^a^R_merge_ = ∑\|I_i_ - I_m_\|/∑I_i_, where Ii is the intensity of the measured reflection and I_m_ is the mean intensity of all symmetry related reflections. | | |
| ^b^*CC*_1/2_ is the correlation coefficient of the half datasets. | | |
| ^c^R_work_ = Σ\|\|F_obs_\| - \|F_calc_\|\|/Σ\|F_obs_\|, where F_obs_ and F_calc_ are observed and calculated structure factors. | | |
| R_free_ = Σ_T_\|\|F_obs_\| - \|F_calc_\|\|/Σ_T_\|F_obs_\|, where T is a test data set of about 5 % of the total reflections randomly chosen and set aside prior to refinement. | | |

**Table B. Summary of plasmids.**

| PLASMID | SOURCE / RRID |
| --- | --- |
| 32m3c-liprin-α2_CC2 (aa 259-542) | This paper |
| pET28a-RIM1α_C2B (aa 1166-1334) | This paper |
| pET28a-RIM1α_C2B (E1198R) | This paper |
| pET28a-RIM1α_C2B (R1201Q) | This paper |
| pET28a-RIM1α_C2B (R1239E) | This paper |
| pET28a-RIM1α_C2B (L1245Q) | This paper |
| pET28a-RIM1α_C2B (Q1247A) | This paper |
| pET28a-RIM1α_C2B (R1236E) | This paper |
| pET28a-RIM1α_C2B (K1240E) | This paper |
| 32m3c-liprin-α2_CC2C (aa 404-542) | This paper |
| 32m3c-liprin-α2_CC2N (aa 300-404) | This paper |
| 32m3c-liprin-α2_CC2N (E337R) | This paper |
| 32m3c-liprin-α2_CC2N (R339E) | This paper |
| 32m3c-liprin-α2_CC2N (E344R) | This paper |
| 32m3c-liprin-α2_CC2N (R346E) | This paper |
| 32m3c-liprin-α2_CC2N (L348F) | This paper |
| 32m3c-liprin-α2_CC2N (A350S) | This paper |
| 32m3c-liprin-α2_CC2N (E379R) | This paper |
| 32m3c-liprin-α2_CC2N (R383A) | This paper |
| 32m3c-liprin-α2_CC2N (E380R) | This paper |
| pET28a-liprin-α2_CC12 (aa 24-542) | Reference [1] |
| pET28a-liprin-α2_CC12 (E344R) | This paper |
| pET28a-liprin-α2_CC12 (R346E) | This paper |
| pET28a-liprin-α2_CC12 (R383A) | This paper |
| m3c-RBP2_(SH3)_3_ (aa 178-252+844-1040) | Reference [2] |
| 32m3c-NCav_CT (aa 2151-2327) | Reference [2] |
| pCAG-FLAG-RIM1 | This paper |
| pCAG-FLAG-ELKS1 | This paper |
| pCAG-FLAG-MBP-3C-Liprin-α2 | This paper |
| pTGFP-Liprin-α2 | Reference [3] |
| pTGFP-Liprin-α2-ΔCC2N (aa 1-299+405-1257) | This paper |
| pTGFP-Liprin-α2-ΔCC2C (aa 1-404+543-1257) | This paper |
| pTGFP-Liprin-α2 (E337R) | This paper |
| pTGFP-Liprin-α2 (R339E) | This paper |
| pTGFP-Liprin-α2 (E344R) | This paper |
| pTGFP-Liprin-α2 (R346E) | This paper |
| pFU-EGFP-Liprin-α2 (WT) | This paper |
| pFU-EGFP-Liprin-α2 (ΔCC2N) | This paper |
| pFU-EGFP-Liprin-α2 (E344R) | This paper |
| pFU-EGFP-Liprin-α2 (R346E) | This paper |
| pFU-NLS-GFP-Cre | Reference [4] |
| pMDLg/pRRE | RRID: Addgene_12251 |
| pRSV-REV | RRID: Addgene_12253 |
| pVSVG | RRID: Addgene_35616 |
| pFU-M2rtTA | RRID: Addgene_20342 |
| pTet-O-Ngn2-puromycin | RRID: Addgene_52047 |
| pFU-oChIEF-tdTomato | Reference [4] |

**Table C. Antibody identifiers.**

| ANTIBODY | SOURCE | IDENTIFIER / RRID |
| --- | --- | --- |
| Mouse anti-β-actin | Sigma | Cat #: A5441; RRID: AB_476744 |
| Rabbit anti-Ca2+ channel P/Q-type alpha-1A | Synaptic Systems | Cat #: 152 203; RRID: AB_2619841 |
| Mouse anti-FLAG | Transgen | Cat #: HT201-01 |
| Mouse anti-GFP | Transgen | Cat #: HT801-01 |
| Rabbit anti-GFP | Thermo Fisher Scientific | Cat #: A11122; RRID: AB_221569 |
| Rabbit anti-Liprin-α2 | Gift from S. Schoch | Zürner et al., 2011 |
| Chicken anti-MAP2 | Encor | Cat #: CPCA-MAP2; RRID: AB_2138173 |
| Mouse anti-PSD95 | Thermo Fisher Scientific | Cat #: MA1-046; RRID: AB_2092361 |
| Mouse anti-PSD95 | NeuroMab | Cat #: 75-028(K28/43); RRID: AB_2877189 |
| Rabbit anti-PSD95 | Addgene | Cat #: 196561(K28/43); RRID: AB_2928071 |
| Rabbit anti-RIM1 | Synaptic Systems | Cat #: 140003; RRID: AB_887774 |
| Rabbit anti-RIM1/2 | Synaptic Systems | Cat #: 140213; RRID: AB_2832237 |
| Rabbit anti-Synapsin | Custom made | Marcó de la Cruz et al., 2024 |
| Mouse anti-Synaptophysin1 | Synaptic Systems | Cat #: 101 011; RRID: AB_887824 |
| Mouse anti-Tuj1 (α-βIII-Tubulin) | BioLegend | Cat #: 801201; RRID: AB_2313773 |
| Horse anti-Mouse HRP | Cell Signaling | Cat #: 7076 |
| Goat anti-Mouse 680RD | LI-COR | Cat #: 925-68070; RRID: AB_2651128 |
| Goat anti-Rabbit 800CW | LI-COR | Cat #: 925-32211; RRID: AB_621843 |
| Goat anti-Mouse Alexa Fluor 488 | Thermo Fisher Scientific | Cat #: A-11001; RRID: AB_2534069 |
| Goat anti-Mouse Alexa Fluor 568 | Thermo Fisher Scientific | Cat #: A-11004; RRID: AB_2534072 |
| Goat anti-Mouse Alexa Fluor 633 | Thermo Fisher Scientific | Cat #: A-21052; RRID: AB_2535719 |
| Goat anti-Rabbit Alexa Fluor 405 | Thermo Fisher Scientific | Cat #: A-31556; RRID: AB_221605 |
| Goat anti-Rabbit Alexa Fluor 488 | Thermo Fisher Scientific | Cat #: A-32731; RRID: AB_2633280 |
| Goat anti-Rabbit Alexa Fluor 568 | Thermo Fisher Scientific | Cat #: A-11011; RRID: AB_143157 |
| Goat anti-Mouse Alexa Fluor 594 | Thermo Fisher Scientific | Cat #: A-11032; RRID: AB_2534091 |
| Goat anti-Rabbit Alexa Fluor 633 | Thermo Fisher Scientific | Cat #: A-21071; RRID: AB_2535732 |
| Goat anti-Rabbit Alexa Fluor 647 | Thermo Fisher Scientific | Cat #: A-21245; RRID: AB_2535813 |
| Goat anti-Chicken-CF405M | Sigma | Cat #: SAB4600466 |
| Goat anti-Chicken Alexa Fluor 633 | Thermo Fisher Scientific | Cat #: A-21103; RRID: AB_2535756 |

**References**

1. Liang M, Jin G, Xie X, Zhang W, Li K, Niu F, et al. Oligomerized liprin-α promotes phase separation of ELKS for compartmentalization of presynaptic active zone proteins. Cell Rep. 2021;34(12):108901. doi: 10.1016/j.celrep.2021.108901. PubMed PMID: 33761347.

2. Wu X, Cai Q, Shen Z, Chen X, Zeng M, Du S, et al. RIM and RIM-BP Form Presynaptic Active-Zone-like Condensates via Phase Separation. Mol Cell. 2019;73(5):971-84.e5. Epub 20190117. doi: 10.1016/j.molcel.2018.12.007. PubMed PMID: 30661983.

3. Xie X, Luo L, Liang M, Zhang W, Zhang T, Yu C, et al. Structural basis of liprin-α-promoted LAR-RPTP clustering for modulation of phosphatase activity. Nat Commun. 2020;11(1):169. Epub 20200110. doi: 10.1038/s41467-019-13949-x. PubMed PMID: 31924785; PubMed Central PMCID: PMCPMC6954185.

4. Marcó de la Cruz B, Campos J, Molinaro A, Xie X, Jin G, Wei Z, et al. Liprin-α proteins are master regulators of human presynapse assembly. Nat Neurosci. 2024;27(4):629-42. Epub 20240312. doi: 10.1038/s41593-024-01592-9. PubMed PMID: 38472649; PubMed Central PMCID: PMCPMC11001580.
