# Supplementary material for: The liprin-α/RIM complex regulates the dynamic assembly of presynaptic active zones via liquid–liquid phase separation
Source: PLoS Biol. 2025 Jun 10;23(6):e3002817. doi: 10.1371/journal.pbio.3002817 (PMC12151379; doi:10.1371/journal.pbio.3002817)

S1 uncropped blot image

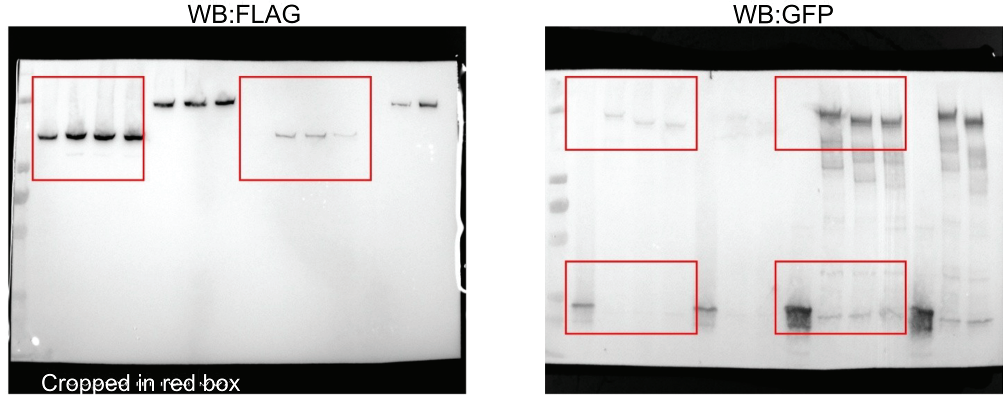

S4E uncropped gel image

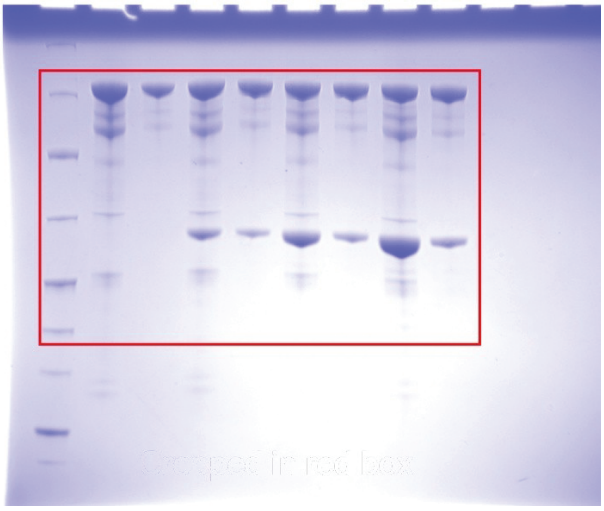

S5A uncropped blot image

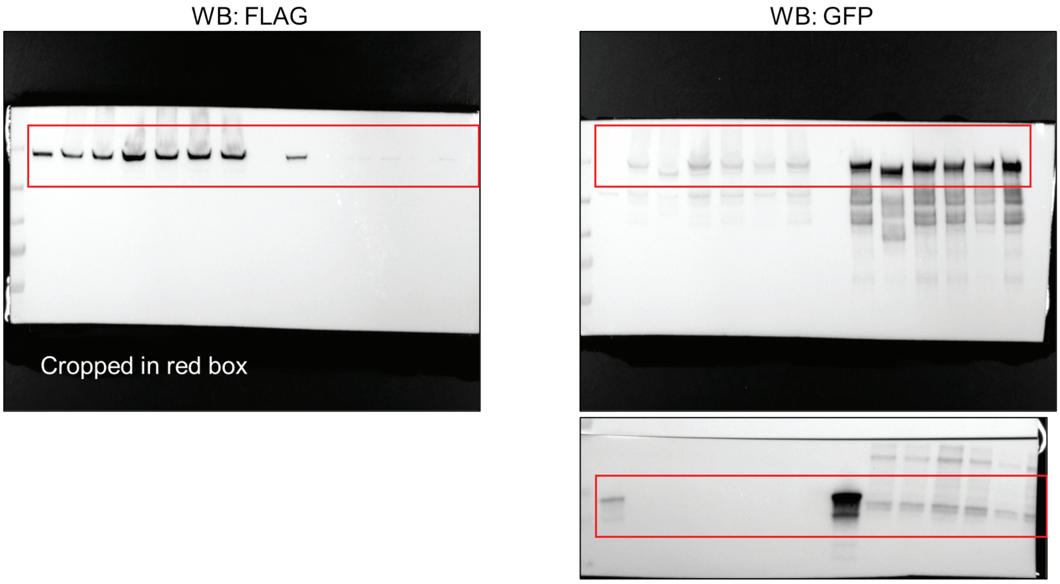

S5B uncropped blot image

WB:FLAG

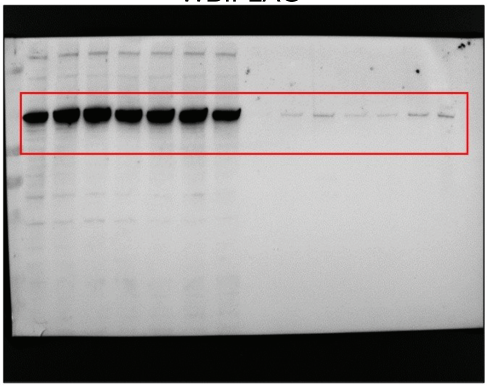

WB:GFP

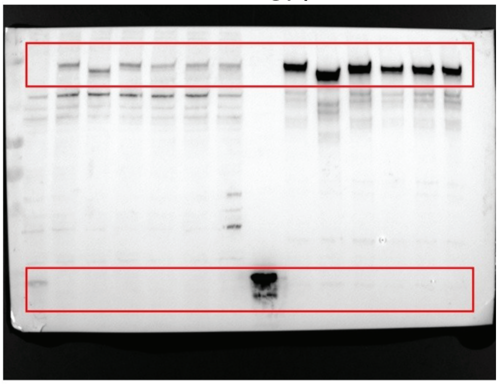

S5C uncropped blot image

WB:GFP

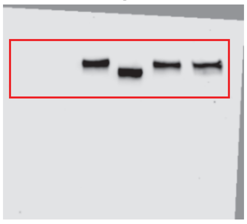

WB:Liprin-α2

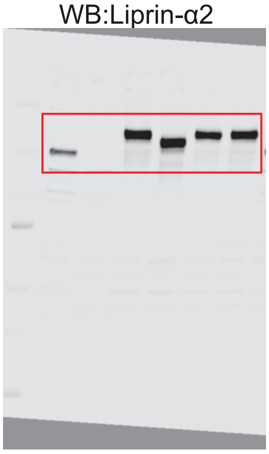

WB:β3-tubulin

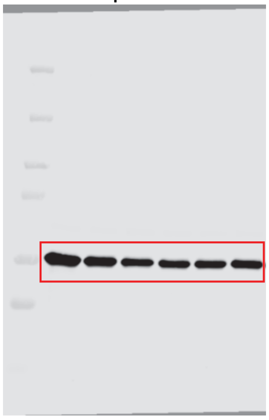

S8A uncropped blot image

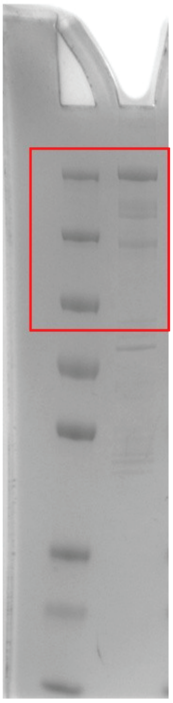

Supplement: S1 Raw Images — (PDF) [file pbio.3002817.s011.pdf]
