# Supplementary material for: The liprin-α/RIM complex regulates the dynamic assembly of presynaptic active zones via liquid–liquid phase separation
Source: PLoS Biol. 2025 Jun 10;23(6):e3002817. doi: 10.1371/journal.pbio.3002817 (PMC12151379; doi:10.1371/journal.pbio.3002817)
Supplement: Attachment — Submitted filename: Response.docx [file pbio.3002817.s014.docx]

**Point-by-point Response**
(Our response is shown in blue)

We sincerely thank all reviewers for acknowledging the novelty and significance of our study and for their critical and constructive comments that helped us improve our manuscript. During revision, we re-analyzed previous electrophysiological datasets to obtain deeper insights, performed additional experiments with purified full-length liprin-α2 to address specific concerns, and toned down multiple parts of the Results and Discussion sections. The updated figures and their respective panels mentioned in our response follow the revised arrangement in the updated manuscript unless otherwise indicated.

Reviewer #1 (Stephan Sigrist, signs review): This study reveals the structure of the complex formed between the coiled-coil region of liprin-α2 and the C2B domain of RIM1, providing new insights into active zone assembly. The authors demonstrate that mutations in liprin-α, associated with neurodevelopmental disorders, block this complex's formation, underlining its importance in synapse development. Structural analysis shows a unique interaction between the coiled-coil and C2B dimers, which drives large protein assemblies and promotes RIM1 condensate formation, confirming liprin-α2's scaffolding role in early active zone protein recruitment.

Using human neurons lacking liprin-α (liprin-α qKOs), the authors found that while the liprin-α/RIM interaction is not required for synapse formation, it is essential for synaptic transmission and vesicle recruitment. Their analyses suggest that liprin-α2, through its interaction with RIM1, not only facilitates RIM1 accumulation at the active zone but also promotes VGCC (voltage-gated calcium channel) clustering. This clustering ensures efficient coupling between VGCC and vesicle priming sites, crucial for effective neurotransmitter release.

General Feedback:

The study offers valuable insights into the structure of the liprin-α/RIM complex, revealing its potential role in active zone assembly. However, certain claims in the paper should be reconsidered for a more measured approach given the in vitro context and the limitations of the physiological data.

We appreciate the positive feedback from Reviewer #1 and the constructive nature of the comments, which we believe have significantly contributed to improving our manuscript. We agree some of the approaches we used, like in any other study, have limitations. We have now included in the discussion where we explicitly mentioned/acknowledged these limitations, as described in detail below. In addition, we have refined both our data presentation and the language used, as detailed in the following responses.

Electrophysiology Approach:

*	While the study is impressive, I am less enthusiastic about the electrophysiology approach, particularly in Figure 5. For example, the authors could have quantified the kinetics of spontaneous release events to assess if fusion kinetics were altered. Relying solely on sucrose treatment is risky when directly correlating it with synaptic features. The extent to which sucrose-triggered release accurately reflects the readily releasable pool (RRP) is debatable, as estimates may vary depending on the sucrose concentration used.

We agree with the reviewer and thank him for these suggestions. Regarding Figure 5, we have now quantified in detail the mEPSC size (amplitude) and kinetics (rise and decay). We did not observe any significant changes in these parameters upon disruption of the liprin-α2/RIM1 interaction. These updated results have been included in the revised Figure 5.

The reviewer pointed out that release triggered by hypertonic sucrose may not accurately reflect the RRP. We agree with this view and thus have toned down our interpretations to acknowledge the limitations of the experimental approach (Lines 289-305). We have also added a note in the discussion (Lines 479-506), emphasizing the need for future experiments using complementary approaches, such as electron microscopy, to conclusively determine the effect of the liprin-α2/RIM1 complex disruption on the RRP size.

*	I am not advocating for additional experiments, given the substantial amount of data already provided. However, the authors should recognize the limitations regarding synaptic function. Their assays capture only a limited set of parameters, such as the lack of access to evoked release kinetics, which could provide a more complete understanding of synaptic dynamics. Additionally, the small expression differences between constructs may have influenced the observed physiological effects, which warrants further consideration.

We acknowledge that our optogenetic approach to measure evoked release has certain limitations. Specifically, the use of presynaptic expression of channelrhodopsin to trigger spikes and transmitter release, precludes accurate measurements of the rise-time kinetics of evoked currents. This is unfortunate, because evoked EPSC kinetics often change (get slower, more unreliable, or both) when the coupling between calcium channels and vesicular calcium sensors is disrupted. We have now explicitly mentioned this limitation (Lines 442-444), as suggested by the reviewer. Although this methodological limitation precluded us from measuring the speed of evoked currents, it did allow measurements of the amplitude of evoked currents, which we used to quantify and compare EGTA sensitivity across different conditions.

Moreover, the reviewer raises an important point that “small expression differences between constructs may have influenced the observed physiological effects”. We fully agree that variations in the expression levels of liprin-α2 WT and mutants could potentially account for the results presented in the original manuscript. To address this possibility, we have performed two experiments. First, we assessed the overall expression levels of liprin-α2 WT and mutants using western blots, which showed that both WT and mutants expressed equally well (Fig. S5C, updated). Second, given that our rescue constructs are GFP-tagged, we quantified GFP fluorescence within Synapsin signals apposed to MAP+ dendrites, as a proxy of local presynaptic protein levels upon transduction with WT and mutant viruses. These measurements also found equal presynaptic expression levels of WT and mutant liprin-α2 (see Fig. R1 below, which has also been incorporated into Fig. S5D in the revised manuscript). Together, these results suggest that differences in the expression levels of WT and mutant liprin-α2 are unlikely to be the primary contributors to the observed structural and physiological phenotypes.

*Figure R1.* *Quantifications of local (presynaptic) expression of different rescue constructs. Left. Representative confocal images. Right. Summary plot of presynaptic GFP intensity upon rescue with different liprin-α2 constructs.*

Statistical Soundness:

*	Quote:

"Indeed, we observed that EGTA-AM more effectively blocked evoked release in liprin-α qKO neurons rescued with the R346E mutation compared to those rescued with the WT construct (Fig. 7C and D). This suggests an increased distance between calcium channels and calcium sensors in the release machinery when the liprin-α/RIM interaction is disrupted."

*	The authors should address whether the observed "more effectively" blocked effect is statistically significant. It's important to provide robust statistical validation to support this conclusion, ensuring that the observed effect is not overstated without proper analysis.

We agree with the reviewer – this is a very important experiment. We conducted this experiment twice (two independent batches), recording a large number of cells (30-40 per condition/genotype) each time. This rendered a grand total of 70-80 cells per condition/genotype. To statistically compare the amplitude of the evoked currents under different conditions (WT and mutant rescues with or without EGTA), we did not assume equal standard deviations (SDs) between the DMSO vs EGTA conditions. This is because EGTA is expected to change the trial-to-trial variability, hence potentially changing the SDs. However, for the comparison between wild-type (WT) and mutant (R346E) rescues under EGTA conditions, we assumed similar SDs. The statistical tests used and the corresponding p-values are as follows. WT rescue DMSO vs WT rescue EGTA: Welch’s test, p=0.0368. MUT rescue DMSO vs MUT EGTA: Welch’s test, p<0.0001. WT EGTA vs MUT EGTA: t-test, p=0.0023. These results have been clearly stated in the revised Results, Figure legends, and supplementary material.

Liprin-α2/RIM1 Complex Formation:

*	Statement: "Liprin-α2 and RIM1 form a large complex through multivalent binding."

Suggestion: The authors should soften this claim to reflect the limitations of the data. A revised phrasing like "Liprin-α2 and RIM1 might form a large complex through multivalent binding" or "Liprin-α2 and RIM1 can form a large complex through multivalent binding in vitro" would more accurately convey the conditional nature of this finding, as it has not been confirmed in vivo.

As suggested, we have revised the sentence as follows: "Liprin-α2 and RIM1 can form a large complex through multivalent binding *in vitro*".

Discussion Language:

*	The phrase "Our study unveils the unique assembly mechanism of the liprin-α/RIM complex, a critical yet less explored aspect," feels somewhat overstated considering the in vitro nature of the work and the physiological study's limitations. A more tempered approach would reflect the context and constraints of the findings more appropriately.

As suggested, we have revised the sentence as follows: "Our study offers insights into the assembly mechanism of the liprin-α/RIM complex, a critical yet less characterized interaction in the presynaptic active zone.”

*	Similarly, the phrase "Remarkably, our discovery of the liprin-α/RIM interaction's role in orchestrating the interplay of the RIM1 and ELKS1 condensates provides fresh insights into the nano-scale organization of the active zone" should be toned down. Given the in vitro context, a more measured statement would better represent the study's impact without overextending the conclusions.

As suggested, we have changed the sentence as follows: "Furthermore, our discovery elucidates the role of the liprin-α/RIM interaction in regulating the interplay between the RIM1 and ELKS1 condensates *in vitro*, providing a plausible explanation for the nano-scale organization of the active zone."

Relevant Recent Work:

*	The authors may want to reference recent work using single-molecule imaging in Drosophila, which demonstrates cooperation between ELKS-type BRP and the Ca²⁺ channel intracellular C-terminal in a compaction transition. Discussing this work could provide useful context and depth to their analysis of the liprin-α/RIM complex.

The paper (Ref. 53) that the reviewer suggested is indeed very important and highly relevant to our study. We apologize for not having carefully discussed the paper in the original version of our manuscript. In that elegant work, Ghelani et al. analyzed the formation and regulation of VGCC nanoclusters in the active zone of drosophila neuromuscular junctions using single-molecule imaging techniques. Interestingly, in our *in vitro* LLPS mixing assay, the liprin-α/RIM interaction confines ELKS1 to the periphery of RIM1 condensates. This spatial organization of ELKS1 results in the central localization of highly concentrated NCav_CT within RIM1 condensates, a pattern resembling the VGCC compaction observed in drosophila neuromuscular junctions. This further supports the potential role of LLPS in the dynamic sub-active zone clustering of VGCC and other active zone proteins in the active zone. We have incorporated a discussion of these findings and their implications in the revised manuscript (Lines 545-552).

Reviewer #2: Jin and colleagues used structure biology tools to understand presynaptic active zone assembly. They determined the crystal structure of a fragment of the liprin-α2/RIM1 complex. They report that neurodevelopmental disease-associated mutations block the formation of the complex. Using some of these mutations, they showed that disrupting this interaction in neurons impairs synaptic transmission and reduces the readily releasable pool of synaptic vesicles. Both Liprin-a and RIM1 are important presynaptic active zone molecules.  This is the first structural paper on the interaction between these two important AZ organizers. The structure part of this manuscript is very high quality and well executed.

However, the synaptic transmission phenotype is quite subtle. They performed in vitro LLPS assays to understand the molecular functions of the liprina2-RIM1 interaction.  They argue that this interaction might recruit Ca channel to active zone. However, several important technical concerns and results challenge this notion.

First, the RIM1 reduction at the active zone in the absence of liprin-α2/RIM1 is quite subtle.

We agree with the reviewer: the reduction in RIM1 levels observed upon the disruption of liprin-α2/RIM1 complexes is subtle. It is likely because, in addition to its interactions with liprin-α, RIM1 interacts with several other presynaptic components (Munc13, RIMBP, calcium channels, etc.) via its additional domains. These additional interactions may partially compensate for the loss of the liprin-α/RIM interaction, facilitating RIM1’s localization to the active zones. Consistently, we observed that the disruption of the liprin-α/RIM interaction results in only a roughly 30% reduction in RIM1 levels at the active zone (Fig. 5G), indicating that under these conditions, RIM1’s functions in vesicle docking, priming, and calcium coupling remain largely preserved. This could potentially explain the lack of more prominent functional/physiological phenotypes in the absence of the liprin-α/RIM interaction.

Second, CaV concentration at AZ is normal, directly arguing against the hypothesis.

Indeed, our STED experiments revealed no changes in the abundance of calcium channels at the active zone upon the disruption of the liprin-α/RIM interaction. However, we do not think that this result argues against our hypothesis, as suggested by the reviewer, but rather supports our premise: namely that liprin-α2/RIM1 controls the clustering/nano-scale organization (as opposed to the abundance) of calcium channels at release sites. A similar phenotype has been previously reported for RBP1/2 knockouts at the calyx of Held synapses (Ref. 46), where direct measurements of presynaptic calcium currents showed no significant differences between RBP control and mutant synapses. Nevertheless, the nano-scale organization of these channels appeared to be disrupted, as indicated by an increased EGTA sensitivity of evoked currents in the absence of RBPs.

Third, the LLPS droplet mixing assay is interesting but its in vivo relevance is unclear.

Given that LLPS is an emerging factor in regulating active zone organization and considering the potential presence of various condensates (*e.g*., liprin-α, ELKS, RIM, and synaptotagmin) in the active zone, it is likely that mesoscale protein-protein interactions coordinate the interplay among these condensates. Understanding the potential consequence of such condensate interactions is therefore intriguing. Due to the technical challenges in manipulating condensates *in vivo*, our LLPS mixing assay, although being more or less artificial, mimics this scenario *in vitro*. The results provide a plausible explanation for the disruptive effects on synaptic transmission caused by the disruption of the liprin-α/RIM interaction. In addition, as Reviewer #1 pointed out, our observations may align with previous *in vivo* observations of the dynamic change of VGCC nanoclusters using single-molecule imaging techniques (Ref. 53). In our LLPS mixing assay (Fig. 6), the liprin-α/RIM interaction confines ELKS1 to the periphery of RIM1 condensates, resulting in the central localization of highly concentrated NCav_CT within RIM1 condensates–a pattern resembling the VGCC compaction observed in drosophila neuromuscular junctions. This further highlights the potential role of LLPS in the dynamic sub-active zone clustering of VGCC and other active zone proteins in the active zone. We have incorporated a related discussion in the revised manuscript (Lines 545-552).

Lastly, almost all experiments were performed only with the CC2 fragments of liprin.  Given that other segments of liprin have been shown to play important roles in synaptic assembly, key results should be repeats using full length constructs.  This is particularly important for the interesting results presented in Fig. 6.

We completely agree with the reviewer that our observations would be more physiologically relevant using full-length liprin-α proteins. To obtain full-length liprin-α2 samples, we tried various expression systems and strategies. Finally, we succeeded in expressing full-length MBP-tagged liprin-α2 in HEK293F cells, which allowed us to purify a sufficient quantity of protein for LLPS assays (Fig. R2A), although the expression efficiency was considerably low. However, our attempts to express this protein with additional GFP or mCherry tags were unsuccessful, and the limited protein yield precluded us from performing efficient fluorescence labeling. Therefore, we cannot directly visualize liprin-α2 in condensates using fluorescent signals. To validate the participation of liprin-α2 in the ELKS condensate formation, we performed LLPS analysis by mixing liprin-α2 with ELKS. Consistent with our previous observations using protein fragments (Ref. 23), the size of ELKS droplets dramatically expanded in the presence of liprin-α2 (Fig. R2B), indicating that ELKS forms co-condensates with liprin-α2.

*Figure R2. Analyses of full-length liprin-α2’s impact on ELKS condensates. (A) SDS-PAGE analysis of purified full-length liprin-α2. (B) Confocal imaging of ELKS droplets with or without liprin-α2.*

Furthermore, to repeat our LLPS results using the full-length protein, we added the ELKS/liprin-α2 co-condensate into pre-formed RIM1/RBP2 condensates. As expected, full-length liprin-α2 effectively restricted the entry of ELKS into RIM/RBP condensates (Fig. R3), comparable to the effect observed with the CC12 fragment (Fig. 6C). This result reinforces our findings based on the CC12 fragment. Additionally, we attempted to obtain full-length mutants. However, despite extensive purification trials, we failed to get any mutant samples of reasonable quality.

*Figure R3.* *Confocal imaging analysis of the LLPS mixture containing the ELKS1/Liprin-α2 co-condensate and the RIM1/RBP2_(CH3)_3_ co-condensate. Magnified views of representative RIM1(a) and ELKS1(b) droplets were displayed below, with line analyses of fluorescence signal intensities along the indicated lines. The concentration of each protein within the mixture was 5 µM.*

These results have been integrated into the Results section (Lines 421-428) of the revised manuscript, and the data presented in Fig. R2 and R3 have been incorporated into a new supplementary figure (Fig. S8).

Specific comments:

1.	In Fig. 4E, the authors used the size of the condensate as a measure for the strength of LLPS.  The condensate size is a function of time.  The longer one wait the bigger condensates will be.  How was the 5 mins incubation time chosen? Since this is a quite artificial condition, it would be more convincing to show a time course of condensate growth instead of only 1 time point.

We thank the reviewer for raising this important point. As the reviewer noted, the condensate size is a function of time. In our experiments, we selected a 20-minute incubation time after comparing condensate growth at different time intervals. We sincerely apologize for our incorrect description of the experimental process. We have corrected this error in the Method and emphasized this time point for image capture in the legend of Figure 4. As shown in Fig. R4 below, after adding the liprin-α2_CC12 variants, the differences in condensate sizes between WT and mutants become most obvious after 20 minutes. As no further significant change in condensate size after 25 minutes, we chose the 20-minute time point for data presentation in Fig. 4E. Additionally, as suggested by the reviewer, we have included this comparison of condensate growth at different time points in Fig. S4D in the revised manuscript.

*Figure R4. The time course of condensate formation following the mixing of RIM1 and liprin-α2_CC12 or its mutants. The concentration of each protein within the mixture was 5 µM.*

2.	If the liprin a2-RIM1 interaction is required for RIM1 recruitment to the active zone, why was there no difference in the data shown in Fig. S6C?  this figure seems to be not consistent with Fig. 5.

The data shown in Fig. S6C is based on confocal images. Due to the spatial resolution limit of confocal microscopy, the quantification shown in Fig. S6C likely reflects the target protein levels for the whole synaptic terminal. However, through STED imaging, a well-established method for quantitative analysis of presynaptic protein levels (Ref. 10 and 39), we could conduct more precise measurements of local RIM1 level changes at the active zone (Fig. 5F). Our data show that disrupting the liprin-α/RIM interaction leads to a ~30% decrease in active zone RIM1 levels. This suggests that while the liprin-α/RIM interaction is dispensable for the synaptic localization of RIM1, it plays a critical role in modulating RIM1 levels at the active zone. Nevertheless, as the confocal quantification of active zone proteins (Fig. S6C) can potentially create confusion, we have decided to remove Fig. S6C&D from the revised manuscript.

3.	The RIM1 reduction shown in Fig.5 is quite moderate. If this small reduction in RIM1 level is responsible for the electrophysiological phenotypes reported in Fig.4, one would predict that heterozygous RIM1 knockout should show even stronger phenotypes.  That should be tested.

Again, we agree that the phenotypes resulting from the disruption of the liprin-α/RIM interaction are moderate. However, these subtle changes are crucial for the clustering of calcium channels at the active zone, as shown in Figures 6 and 7. We also agree with the reviewer that if the reduction in active zone RIM1 fully accounted for the observed phenotypes, haploinsufficiency of RIM1 would be expected to render similar physiological phenotypes. Indeed, we have performed this experiment in a project outside the scope of the current manuscript (see Fig. R5 below), which we are happy to share with the reviewer.

We generated heterozygous RIM1 knockout hESC lines with CRISPR/Cas9, using a double-guide approach as shown in Fig. R5A. Then we differentiated these clones into pure excitatory neurons by forced expression of Neurogenin2 and confirmed a nearly 50% reduction in RIM1 levels using western blot (Fig. R5B). We then measured transmission and observed a sizeable reduction in the frequency of mEPSCs. Last, using high-pressure freezing EM, we found that the number of synaptic vesicles in very close apposition to presynaptic membranes (likely a morphological correlate of primed synaptic vesicles) was reduced in RIM1 heterozygous KO neurons (Fig. R5C). Altogether, these results strongly suggest that the partial loss of local RIM1 from the active zones can indeed account for the electrophysiological phenotypes presented in Fig. 4. We hope the reviewer concurs.

*Figure R5. Synaptic and morphological phenotypes upon deletion of a single copy of RIM1. A. strategy used to generate RIM1 heterozygous KO lines (top), and sequence of guide used (bottom). B. western blot analysis of putative Het clones. C. summary plots of mEPSC frequency (left) and number of docked vesicles (right) in RIM1 control and heterozygous KO neurons.*

4.	Fig. 6 used a large number of in vitro experiments to suggest that the CC12-RIM interaction help to recruit Ca channels to the condensate.  However, in Fig. 5, the direct measurements of CaV level at presynaptic terminal showed no defects.  This is an important question because of the highly artificial nature of the droplet experiments.  Without in vivo validation, it calls the relevance into question.

The results presented in Fig. 6 are consistent with the idea that liprin-α2/RIM1 complexes play a role in recruiting calcium channels to the release sites. As release sites are nano-domains within the active zone, these *in vitro* results support our hypothesis that the liprin-α/RIM interaction fine-tunes the nanoscale organization of calcium channels within the active zones. Although our STED experiments (Fig. 5) indicate that the abundance of calcium channels at the active zone is not affected, we proposed that their clustering, distribution, and more precisely, their physical distance to the vesicular calcium sensor Synaptotagmin-1 is impaired. This is further supported by our EGTA experiment (Fig. 7), which in our view, provides direct evidence for this claim in actual mammalian synapses.

5.	Data presented in Fig. 7 suggests that there are defects in the coupling between Ca channels and release machinery. To reconcile this result with the lack of CaV defects in Fig. 5, it is possible that there are sub-active zone distribution defects.  However, these difference needs to be measured.  For example, if RIM, ELKS1 and RBP show differential sub active zone localization, it would be an exciting hypothesis that these "AZ" scaffolds are organized into non uniform clusters.  At this point, the EGTA experiments do not directly show that.

We fully agree with the reviewer that our data support the presence of sub-active zone defects upon the disruption of the liprin-α/RIM interaction. In our view, this is exactly what Fig. 6 and 7 combined suggest. We also agree that assessing the sub-active zone distribution of RIM, ELK, RBP, and calcium channels in human neurons is an exciting and important idea. Such kind of analysis would require the use of state-of-the-art STORM or single-particle PALM microscopy. All these exciting experiments, however, are beyond the immediate scope of this manuscript, and we anticipate exploring them in future projects. We have now included a statement in the discussion, addressing these views (Lines 510-517).

Reviewer #3 (Da Jia, signs review): Neurotransmitters are released by synaptic vesicle exocytosis at the active zone of a presynaptic nerve terminal. Action potential (AP) induces presynaptic membrane depolarization and subsequent opening of Ca2+ channels, and then triggers neurotransmitter release at the active zone of presynaptic terminathe. In this progress, active zone lies at the interface between the presynaptic terminal and the synaptic cleft, and transform a presynaptic action potential signal into a released neurotransmitter signal. This manuscript by Jin et al. describes the structure of the liprin-α2_CC2N/RIM1_C2B complex, and unveils the unique assembly mechanism of the liprin-α/RIM complex in the presynaptic active zone. The study is of overall high quality and interest. I only have a few suggestions that could future elevate the significance of the study.

1. In Fig.2F, please show the kd values of C2N and C2B.

Following the reviewer’s suggestion, we have added the K_d_ value of CC2N and C2B as a reference in Fig. 2F.

2. In Fig.3F, A350liprin-α2 didn't directly involved in C2B binding, but contributes to the coiled-coil formation of CC2. Does the A350S mutant alter the LLPS property?

We thank the reviewer for reminding us about the possibility that the A350S mutation might alter the role of liprin-α2 in promoting RIM1 LLPS. Although A350 is involved in the coiled-coil formation, the A350S mutation does not appear to either disrupt or enhance the CC2 structure (Fig. 3E), supported by the unchanged purification profile of the A350S CC2 mutant and its similar binding affinity to C2B. Since this mutation affects little self-association/protein binding, in our view, the presence of the A350S mutation in liprin-α2 would not affect the LLPS property of RIM1.

3. Interestingly, Jin et al purified RIM1_C2B was a monomer, while it forms a homodimer in their crystal structure. Which amino acids mediate the formation of dimeric interfaces? Do these amino acids alter the LLPS property?

We thank the reviewer for raising this point. In our manuscript, we have not extensively analyzed the dimerization interface of RIM1_C2B, as this aspect has already been thoroughly described in the apo structure of RIM1_C2B (Ref. 38). Both terminal regions and a part of β-sheets of the C2B domain contribute to the dimer formation (Fig. 4A, also see Fig. R6 below). As we have proposed in the manuscript, the dimer formation of C2B may potentially promote the assembly of a large complex between liprin-α2 and RIM1 by providing multivalent interactions (Fig. 4C), which consequently enhances RIM1 LLPS. It is conceivable that disrupting C2B dimerization could lead to a reduction of RIM1 LLPS. To test this possibility, we have designed the Q1262A mutation to disrupt the dimer interface (Fig. R6). However, the poor quality (heavy aggregation) of the mutant RIM1 protein, possibly due to the problem of local folding, prevented further analysis.

*Figure R6. The RIM1_C2B dimer structure. In the dimer interface, Q1262 forms two hydrogen bonds with the backbone atoms in a loop of the C2B C-terminal region.*

Reviewer #4 (Drago Milovanovic, signs review): In the submitted manuscript, Jin and colleagues present a detailed mechanistic insight into the roles of the liprin-α/RIM-1 complex in the regulation of calcium channel dynamics and synaptic vesicle release. The manuscript spans across scales from the nanoscale crystal structure and characterization of liprin-α (CC2N region)/RIM-1 (C2B domain); to mesoscale formation of condensates showing the liprin-α/RIM-1 complexes regulate the distribution of ELKS1 and VGCC; to the functional impact assessed in the iPSC-derived human neurons lacking all liprin-α proteins (liprin-α qKO neurons). This is a carefully designed, thoroughly executed study that puts forward an exemplary role of how the liprin-α/RIM-1 interactions, while being dispensable for the assembly of the active zone, play a major role in fine-tuning the primed synaptic vesicles (SVs) and neuronal transmission. Therefore, I strongly endorse its publication with a few suggestions to address:

(i) The assembly of the active zone in liprin-α qKO neurons rescued with mutants that prevent the interactions between liprin-α/RIM-1 seems to be unaltered (Fig. S6). Yet, the evoked release in liprin-α qKO neurons rescued with either liprin-α WT or liprin-α (R346E/unable to make a complex with RIM-1) was significantly different (Fig. 7). How do authors ensure that R346E doesn't lead to misaligned or unspecialized synapses, which may lack a functional postsynapse? Is the frequency of fully mature pre-/post-synaptic assemblies the same in these different rescue experiments? Control staining with a postsynaptic marker would ensure that in rescue experiments of liprin-α qKO neurons, there is a proper apposition of the postsynaptic density with the presynaptic terminal.

Great point. We fully agree that the disruption of the liprin-α/RIM1 interaction does not have a major impact on the integrity of the active zone, but rather affects its fine-scale organization. The reviewer asked whether the rescue with R346E mutants might lead to misaligned or unspecialized synapses. We would like to raise two points to address this concern.

1) As suggested by the reviewer, we have quantified the frequency of synapses defined as appositions between synapsin and PSD95 (pre-post junctions). Specifically, for this, we used low-magnification STED images, which we commonly acquire at the beginning of the experiment and routinely use to find synapse-rich areas for high-magnification STED (Fig. S6E). As shown in Fig. R7 below, there was no significant difference in the frequency of fully assembled synapses (defined as appositions between synapsin and PSD95) for WT and mutant rescues.

*Figure R7.* *Quantifications of the density pre-post junctions upon rescue with liprin-α2 WT and R346E. Rescue with mutant liprin-α2 does not seem to affect the frequency of pre-post junctions. n.s, non-significant.*

2) We would like to emphasize that all STED measurements (calcium channels, RIM1) were performed in synapses defined by synapsin-PSD95 apposition – thus on fully assembled pre-post junctions (Fig. 5G and 5H). We apologize for any ambiguity in the previous version of our manuscript regarding this critical detail, which we have further clarified in the revised manuscript (Lines 315-316). Thus, the observed reduction in RIM1 levels is not because some miss-aligned/unspecified synapses were quantified. Instead, it supports our hypothesis that the liprin-α/RIM1 interaction fine-tunes the molecular composition of fully assembled synapses.

(ii) Given that liprin-α/RIM-1 complex regulates the distribution of proteins and vesicles in the active zone/RIM-1 condensates, the authors should discuss this in the context of synapsin/SV phase at the synapse, which ensures the rapid SV motility and accessibility of vesicles within presynapse (PMID: 37872159), as well as the short-distance vesicle transport proposed to channel SVs from the synapsin/SV condensates to the active zone (PMID: 38552623).

We thank the reviewer for the suggestion. We have cited these important papers and extended our discussion concerning various presynaptic condensates in the revised manuscript (Lines 564-570).

(iii) Given the relevance of C2B domains within a range of presynaptic proteins, the authors should also discuss the influence that liprin-α/RIM-1 complexes may have on the recently reported condensation of synaptotagmin-1 (PMID: 38177243 and PMID: 38980206).

As suggested, we have now cited these papers accordingly and included a related discussion (Lines 587-591).
